# Allergy immunotherapy across the life cycle to promote active and healthy ageing: from research to policies: An AIRWAYS Integrated Care Pathways (ICPs) programme item (Action Plan B3 of the European Innovation Partnership on active and healthy ageing) and the Global Alliance against Chronic Respiratory Diseases (GARD), a World Health Organization GARD research demonstration project

**DOI:** 10.1186/s13601-016-0131-x

**Published:** 2016-11-23

**Authors:** M. A. Calderon, P. Demoly, T. Casale, C. A. Akdis, C. Bachert, M. Bewick, B. M. Bilò, B. Bohle, S. Bonini, A. Bush, D. P. Caimmi, G. W. Canonica, V. Cardona, A. M. Chiriac, L. Cox, A. Custovic, F. De Blay, P. Devillier, A. Didier, G. Di Lorenzo, G. Du Toit, S. R. Durham, P. Eng, A. Fiocchi, A. T. Fox, R. Gerth van Wijk, R. M. Gomez, T. Haathela, S. Halken, P. W. Hellings, L. Jacobsen, J. Just, L. K. Tanno, J. Kleine-Tebbe, L. Klimek, E. F. Knol, P. Kuna, D. E. Larenas-Linnemann, A. Linneberg, M. Matricardi, H. J. Malling, R. Moesges, J. Mullol, A. Muraro, N. Papadopoulos, G. Passalacqua, E. Pastorello, O. Pfaar, D. Price, P. Rodriguez del Rio, R. Ruëff, B. Samolinski, G. K. Scadding, G. Senti, M. H. Shamji, A. Sheikh, J. C. Sisul, D. Sole, G. J. Sturm, A. Tabar, R. Van Ree, M. T. Ventura, C. Vidal, E. M. Varga, M. Worm, T. Zuberbier, J. Bousquet

**Affiliations:** 1 National Heart and Lung Institute, Royal Brompton Hospital NHS, Imperial College London, London, UK; 2 Unité d’allergologie, Département de Pneumologie et AddictologieHôpital Arnaud de Villeneuve, CHRU de Montpellier, Sorbonne Universités, UPMC Paris 06, UMR-S 1136, IPLESP, Equipe EPAR, 75013 Paris, France; 3 University of South Florida Morsani College of Medicine, Tampa, FL USA; 4 Christine Kühne Center for Allergy Research and Education (CK-CARE), Swiss Institute of Allergy and Asthma Research (SIAF)University of Zurich, Davos, Switzerland; 5 Upper Airways Research Laboratory (URL), ENT Department, University Hospital Ghent, Ghent, Belgium; 6 iQ4U consultants Ltd, London, UK; 7 Allergy Unit, Department of Internal Medicine, University Hosp Ospedali Riuniti, Ancona, Italy; 8 Department of Pathophysiology and Allergy Research, Center of Pathophysiology, Infectiology and Immunology, Medical University of Vienna, Vienna, Austria; 9 Second University of Naples and IFT-CNR, Rome, Italy; 10 Allergy and Respiratory Diseases Clinic, DIMI, University of Genoa, IRCCS AOU San Martino-IST, Genoa, Italy; 11 Allergy Section, Department of Internal Medicine, Hospital Universitari Vall d’Hebron, Barcelona, Spain; 12 Division of Allergy, Hôpital Arnaud de Villeneuve, Department of Pulmonology, University Hospital of Montpellier, Montpellier - UPMC Univ Paris 06, UMRS 1136, Equipe - EPAR - IPLESP, Sorbonne Universités, Paris, France; 13 Nova Southeastern University, Ft. Lauderdale, FL USA; 14 Allergy Division, Chest Disease Department, University Hospital of Strasbourg, Strasbourg, France; 15 University Versailles Saint-Quentin and Clinical Pharmacology Unit, UPRES EA 220, Department of Airway Diseases, Foch Hospital, Suresnes, France; 16 Respiratory Diseases Department, Rangueil-Larrey Hospital, Toulouse, France; 17 Dipartimento BioMedico di Medicina Interna e Specialistica (Di.Bi.M.I.S), University of Palermo, Palermo, Italy; 18 Guy’s and St. Thomas’ NHS Trust, Kings College, London, UK; 19 Allergy and Clinical Immunology Section, National Heart and Lung Institute, Imperial College London, London, UK; 20 Department of Pediatric Pulmonology and Allergy, Children’s Hospital, Aarau, Switzerland; 21 Division of Allergy, Department of Pediatrics, Bambino Gesù Pediatric Hospital, Vatican City, Rome, Italy; 22 King’s College London Allergy Academy, London, UK; 23 Section of Allergology, Department of Internal Medicine, Erasmus Medical Center, Building Rochussenstraat, Rotterdam, The Netherlands; 24 Unidad Alergia and Asma, Hospital San Bernardo, Salta, Argentina; 25 Skin and Allergy Hospital, Helsinki University Hospital, Helsinki, Finland; 26 Hans Christian Andersen Children’s Hospital, Odense University Hospital, Odense, Denmark; 27 Clinical Department of Otorhinolaryngology, Head and Neck Surgery, University Hospitals Leuven, KU Leuven, Louvain, Belgium; 28 Allergy Learning and Consulting, Secretary Immunotherapy Interest Group EAACI, Copenhagen, Denmark; 29 Allergology Department, Centre de l’Asthme et des Allergies, Hôpital d’Enfants Armand-Trousseau, INSERM, UMR_S 1136, Sorbonne Universités, UPMC Univ Paris, Institut Pierre Louis d’Epidémiologie et de Santé Publique, Equipe EPAR, Paris, France; 30 Hospital Sírio Libanês, São Paulo, Brazil; 31 University Hospital of Montpellier, Montpellier, France; 32 UPMC Paris 06, UMR-S 1136, IPLESP, Equipe EPAR, Sorbonne Universités, Paris, France; 33 Allergy and Asthma Center Westend, Outpatient Clinic and Clinical Research Center, Ackermann, Hanf, & Kleine-Tebbe, Berlin, Germany; 34 Center for Rhinology and Allergology, German Society for Otorhinolaryngology HNS, Wiesbaden, Germany; 35 Departments of Immunology and Dermatology/Allergology, University Medical Center Utrecht, Heidelberglaan, Utrecht, The Netherlands; 36 Medical University of Lodz, Lodz, Poland; 37 ARIA, Mexico, DF Mexico; 38 AAAAI, Hospital Médica Sur, Mexico, DF Mexico; 39 Research Centre for Prevention and Health, The Capital Region of Denmark, Copenhagen, Denmark; 40 Department of Clinical Experimental Research, Rigshospitalet, Copenhagen, Denmark; 41 Department of Clinical Medicine, Faculty of Health and Medical Sciences, University of Copenhagen, Copenhagen, Denmark; 42 Pediatric Pneumology and Immunology, Charité Medical University, Berlin, Germany; 43 Danish Allergy Centre, Allergy Clinic, Gentofte University Hospital, Hellerup, Denmark; 44 IMSIE, Klinikum der Universität zu Köln A. ö. R., Cologne, Germany; 45 Unitat de Rinologia i Clínica de l’Olfacte, ENT Department, Hospital Clínic, Clinical and Experimental Respiratory Immunoallergy, IDIBAPS, CIBERES, Barcelona, Catalonia Spain; 46 Department of Women and Child Health, Food Allergy Referral Centre Veneto Region, Padua General University Hospital, Padua, Italy; 47 Allergy Unit, 2nd Pediatric Clinic, University of Athens, Athens, Greece; 48 Allergy and Respiratory Diseases, IRCCS San Martino-IST, Univesity of Genoa, Genoa, Italy; 49 ASST Grande Ospedale Metropolitano Niguarda, P.zza Ospedale Maggiore, Milan, Italy; 50 Department of Otorhinolaryngology, Head and Neck Surgery, Universitätsmedizin Mannheim, Mannheim, Germany; 51 Medical Faculty Mannheim, Heidelberg University, Heidelberg, Germany; 52 Center for Rhinology and Allergology, Wiesbaden, Germany; 53 Division of Applied Health Sciences, Primary Care Respiratory Medicine, Academic Primary Care, University of Aberdeen, Aberdeen, Scotland, UK; 54 Research in Real Life (RiRL), Oakington, Cambridge, UK; 55 Optimum Patient Care Ltd, Singapore, Singapore; 56 Allergy Section, Hospital Infantil Universitario Niño Jesús, Madrid, Spain; 57 Department of Dermatology and Allergology, Ludwig-Maximillian University, Munich, Germany; 58 Department of Prevention of Environmental Hazards and Allergology, Medical University of Warsaw, Warsaw, Poland; 59 Royal National Throat, Nose and Ear Hospital, London, UK; 60 University College London, London, UK; 61 Clinical Trials Center, University Hospital of Zurich, Zurich, Switzerland; 62 Immunomodulation and Tolerance Group, Allergy and Clinical Immunology, Inflammation, Repair and Development Section, National Heart and Lung Institute, Faculty of Medicine, Imperial College London, London, UK; 63 MRC and Asthma UK Centre in Allergic Mechanisms of Asthma, London, UK; 64 Asthma UK Centre for Applied Research, Centre for Medical Informatics, Usher Institute of Population Health Sciences and Informatics, The University of Edinburgh, Teviot Place, Edinburgh, EH8 9AG UK; 65 SLAAI, Asuncion, Paraguay; 66 Programa de Pòs-Graduação em Pediatria e Ciências Aplicadas à Pediatria, Departamento de Pediatria EPM, UNIFESP, São Paulo, Brazil; 67 Department of Dermatology and Venerology, Medical University of Graz, Graz, Austria; 68 Allergy Outpatient Clinic Reumannplatz, Vienna, Austria; 69 Servicio de Alergologia, Complejo Hospitalario de Navarra, Pamplona, Spain; 70 Departments of Experimental Immunology and Otorhinolaryngology, Academic Medical Center, University of Amsterdam, Amsterdam, The Netherlands; 71 Unit of Geriatric Immunoallergology, Interdisciplinary Department of Medicine, University of Bari Medical School, Bari, Italy; 72 Allergy Department, Complejo Hospitalario Universitario de Santiago de Compostela, Santiago de Compostela, Spain; 73 Respiratory and Allergic Disease Division, Department of Paediatrics, Medical University of Graz, Graz, Austria; 74 Allergie-Centrum-Charité, Klinik für Dermatologie, Venerologie und Allergologie, Charité-Universitätsmedizin Berlin, Berlin, Germany; 75 Contre les MAladies Chroniques pour un VIeillissement Actif en Languedoc-Roussillon, European Innovation Partnership on Active and Healthy Ageing Reference Site, Paris, France; 76 INSERM, VIMA, U1168, Ageing and Chronic Diseases, Epidemiological and Public Health Approaches, Paris, France; 77 UVSQ, UMR-S 1168, Université Versailles St-Quentin-en-Yvelines, Versailles Cedex, France; 78 CHRU, 371 Avenue du Doyen Gaston Giraud, 34295 Montpellier Cedex 5, France

**Keywords:** Allergen immunotherapy, EIP on AHA, AIRWAYS ICPs, Rhinitis, Asthma, Ageing

## Abstract

Allergic diseases often occur early in life and persist throughout life. This life-course perspective should be considered in allergen immunotherapy. In particular it is essential to understand whether this al treatment may be used in old age adults. The current paper was developed by a working group of AIRWAYS integrated care pathways for airways diseases, the model of chronic respiratory diseases of the European Innovation Partnership on active and healthy ageing (DG CONNECT and DG Santé). It considered (1) the political background, (2) the rationale for allergen immunotherapy across the life cycle, (3) the unmet needs for the treatment, in particular in preschool children and old age adults, (4) the strategic framework and the practical approach to synergize current initiatives in allergen immunotherapy, its mechanisms and the concept of active and healthy ageing.

## Background

Allergic diseases often occur early in life and persist throughout life. This life-course perspective should be considered in allergen immunotherapy (AIT). In particular it is essential to understand whether this immunological treatment may be used in old age adults.

The current paper was developed by a working group of AIRWAYS integrated care pathways (ICPs for airways diseases), the model of chronic respiratory diseases of the European Innovation Partnership on active and healthy ageing (DG CONNECT and DG Santé) to develop the concept of AIT across the life cycle and propose a strategic framework to be tested.

## AIRWAYS integrated care pathways

### The political background

Active and healthy ageing (AHA) is a major societal challenge, common to all populations. Ageing, together with gender, socio-economic and other forms of inequalities (https://www.equalityhumanrights.com/en/equality-act/protected-characteristics), is an under-appreciated cause of poor health, which can have an adverse impact on economic development [1]. To tackle the burden of ageing in the European Union (EU), the European Commission launched the European Innovation Partnership (EIP) on AHA within its innovation policy [2]. The EIP on AHA is proposing a novel approach [2] that may be of great benefit to the economic consequences of the demographic changes in Poland. EIPs aim to enhance EU competitiveness and tackle societal challenges through research and innovation. They will address weaknesses in the EU research and innovation (e.g. under-investment, fragmentation and duplication), which complicate the discovery or exploitation of knowledge and may ultimately prevent the entry of innovations into the market place. EIP on AHA pursues a triple win for Europe:

- To enable EU citizens to lead healthy, active and independent lives while ageing.
- To improve the sustainability and efficiency of social and health care systems.
- To boost and improve the competitiveness of the markets for innovative products and services, responding to the ageing challenge at both EU and global level, thus creating new opportunities for businesses.

ICPs for chronic respiratory diseases (AIRWAYS ICPs) have been selected as a model for Action Plan B3 of the EIP on AHA Strategic Implementation Plan [3, 4]. The goals of AIRWAYS ICPs are to launch a collaboration to develop multi-sectoral care pathways for chronic respiratory diseases in European countries, regions and beyond in association with the World Health Organization (WHO) Global Alliance against chronic Respiratory Diseases (GARD research demonstration project) [5]. There are several ongoing actions among which:

- A synergy paper in press.
- A twinning for rhinitis in the elderly accepted yesterday by the EU.
- An App and a tablet for rhinitis and asthma deployed in 20 countries which received funding from private sources and the EU Development and Structural Funds [6–10].
- The scaling up strategy [11].

Prenatal and early-life events play a fundamental role in health and on the development of non-communicable diseases (NCDs) and AHA [12]. The Polish Presidency of the EU Council targeted chronic respiratory diseases in children to promote AHA [13]. It has been recognized that the prevention and early diagnosis and treatment of chronic respiratory diseases have a positive impact on child development and health-related quality of life and markedly contribute to an active and healthy childhood as well as AHA. Therefore, the development of new tools has been proposed for the early recognition and improvement of treatment for these conditions.

### Respiratory allergies across the life cycle

IgE-mediated allergic diseases were defined by the World Allergy Organization [14] and include allergic rhinitis [15], allergic asthma [16], atopic dermatitis (AD) [17] and food allergy. However, IgE-mediated allergy is not always involved in the symptoms of these diseases [18–21] including non-allergic rhinitis, and non-allergic asthma.

Respiratory allergic diseases (asthma and rhinoconjunctivitis) are amongst the most common diseases worldwide [16]. They affect all age groups and rank first in Europe. The burden of these allergic diseases is substantial [22, 23] since they often impair social life as well as school and work performances [15, 24, 25]. Many patients are untreated or uncontrolled (despite treatment) [26], remain under-diagnosed and often do not receive adequate treatment. These factors all contribute to poor disease control.

Allergic diseases often start early in life and tend to persist across the life cycle, from infancy to the elderly. This is especially true with asthma or rhinitis, which may cause problems in individuals over 65 years of age [27]. Longitudinal birth cohort studies have shown sequential events leading to upper and lower respiratory allergies [28, 29]. They start with weak sensitizations to a limited number of allergen components followed by strong and/or new sensitizations to varying allergen sources depending on the region of the world [30–34]. However, in a large number of patients, sensitization does not change over time and is fully developed in pre-school children [29, 32, 35]. Moreover, the allergic march is uncommon [36]. The escalation of sensitization parallels with the clinical expression of the allergic disease. Genuine sensitizers (either for food- or inhalant-related allergens) tend to appear early in life, whereas specific IgE to pan-allergens appear later [37].

Cohort studies following participants into middle age have indicated that several factors, including childhood asthma, low or impaired lung function at an early age as well as atopy are risk factors for chronic obstructive pulmonary disease (COPD) [38, 39]. In asthma, allergic sensitization was associated with worsening of health-related quality of life [40] in the elderly, with a high mortality risk due to under-diagnosis, under-treatment and comorbidity [41]. First-time exposure to an occupational allergen in any context (e.g. during apprenticeship) is indeed a period of increased risk of developing work-related respiratory allergic diseases such as asthma and allergic rhinitis [42]. For example, occupational cleaners are associated with an increased risk of developing both asthma and self-reported COPD [43]. Established occupational asthma may persist in spite of complete allergen avoidance.

Over the past five decades, an “epidemic” of allergic diseases and asthma has been observed globally in children and adults [5]. The expected epidemic wave of asthma and rhinitis in elderly adults remains an insufficiently recognized problem [44–46]. In Europe, over 20% of adults suffer from allergic rhinitis and over 5% from asthma [15, 47]. These patients are now reaching the age of 65 and a new health challenge in elderly people will be to understand, detect and manage them. The importance of AIT in these patients needs to be better defined, tested and included in health policy making for the elderly [13].

Since we are entering an era of rapidly increasing elderly people, and considering that asthma and rhinitis usually commence during childhood or adulthood, both diseases represent an emerging public health concern in all age populations [48, 49].

## Rationale of allergy immunotherapy across the life cycle in AIRWAYS ICPs

### Allergy and allergen immunotherapy scope

Immunotherapy for IgE-mediated allergic diseases, referred to as AIT, has entered a new era [50–53]. It is characterized by: (1) thorough clinical development programmes leading to full registration and standardization of products; (2) better understanding of the functions of the immune system; (3) development of new products and routes to improve efficacy and safety; and (4) harmonized clinical practice parameters based on both evidence-based medicine and clinical experience. AIT includes both allergen- and non-allergen-containing therapeutics and is fully supported by the Polish Presidency of the Council of the EU [13] and the Finnish Allergy Plan [27, 54]. Although dosing is now established for some major allergens [55, 56], mixing of allergens is still a matter of debate and the selection of the allergen in some polysensitized patients may still be difficult.

### AIT induces immune tolerance

The goals for AIT are to control the symptoms of patients who are unable to cope with their disease using optimal pharmacotherapy [9, 57, 58]. Ultimately, AIT aims to induce immune tolerance so that after its discontinuation, there are persistent therapeutic benefits [59, 60]. Whereas the early effects of AIT are probably related to basophil/mast cell down-regulation, AIT produces an early and transient inflammatory cell down-regulation and a transient to long-lasting T cell tolerance [61, 62]. This “tolerance” response is mainly mediated by IL-10, TGF-β and other suppressive factors causing immune deviation towards a more balanced T regulatory cell response that leads to the observed suppression of cytokines from allergen-specific Th1 and Th2 cells [26, 63]. AIT also induces allergen-specific IL-10-producing memory B regulatory cells, which seem to be responsible for specific IgG4 production. Furthermore, AIT is associated with the suppression of total IgE, allergen-specific IgE, increases in allergen-specific blocking IgG_4_ antibodies [64, 65], a decrease in both tissue mast cells and eosinophil numbers [66] and the degree of activation.

### AIT efficacy and safety

Clinical efficacy and safety of AIT for allergic asthma and allergic rhinoconjunctivitis for the most relevant allergen sources have been documented in well-powered randomized controlled trials, at least for specific products. Different systematic reviews and meta-analyses of studies in adults and children have confirmed efficacy for many relevant allergen sources [67–71]. However, some reviews were critical about AIT although certain criticisms were raised [72]. AIT improves disease-specific quality of life [73, 74]. Follow-up studies after the discontinuation of AIT have demonstrated a carry-over effect which can last for up to 12 years after its cessation [69, 75], but these studies are hampered by a high rate of drop outs and the effectiveness of these long-lasting effects needs more attention. AIT is thus a disease-modifying intervention (the only one currently available) with the potential of altering the allergic march [76, 77]. Double blind randomized controlled studies are currently ongoing in children allergic to grass pollens [78] and house dust mite to assess the potential prevention of asthma (EudraCT 2012-005678-76) (Table 1). It has been suggested that AIT is cost-effective although more information is needed [53, 79, 80].

**Table 1:** Types of biomarkers for AIT

Identification and validation of biomarkers assessing the probability of response to treatment of AIT before it is initiated
Identification and validation of biomarkers assessing the safety of AIT before it is initiated
Identification and validation of biomarkers confirming the efficacy of AIT in patients receiving AIT (short and long term)
Identification and validation of biomarkers predicting the long-term effects of AIT before it is stopped
Identification and validation of biomarkers predicting the relapse of symptoms when AIT is stopped

### Unmet needs

1. *Tolerance to AIT in very young children* Considering that children born to atopic parents are at considerable increased risk of sensitization to common environmental allergens [81], it is important to establish the possible preventive role of AIT in reducing sensitization (primary prevention) to any allergens when given at infancy although the first results are inconclusive [82, 83] or difficult to interpret [84].
2. *When to start AIT in preschool children* The demonstration of a progressive molecular-spreading process in allergic rhinitis [30] has provided the rationale for an early allergen-specific immunological intervention [85]. In this respect, studies on a ‘secondary allergen immuno-prophylaxis’ may be targeted to children who are already sensitized to airborne allergens but still asymptomatic. The on-going allergic immune response is likely to be more susceptible of being suppressed in the first, weaker, pre-clinical monomolecular stages, rather than in the polymolecular clinical stages. It is also plausible that AIT will have a greater efficacy if started immediately after the disease onset rather than, as usual, years later. This concept of ‘early AIT’ should be tested in properly designed prospective trials [85].
  The results of the EU programme on Mechanisms of the Development of ALLergy (MeDALL) have improved the stratification of allergic preschool children for diagnosis, prognosis and AIT [28, 29]. Multi-morbidity, co-existing allergic diseases (such as atopic eczema, allergic rhinitis and asthma) [86] and/or IgE polysensitisation are markers of clinical disease and disease persistence. For the first time ever, MeDALL is proposing a scientific answer for the initiation of AIT in pre-school children as it has found that the vast majority of those with multi-morbidities will have persistent allergic conditions [33, 87]. This finding has the potential to guide physicians to consider starting AIT early in life and this might lead to a greater expected efficacy to modify the course of allergy. Moreover, allergy with polysensitisation to birch pollen [32], cat or dog [31], or peanut (Asarnoj, in preparation) at age four can predict the onset and/or persistence of rhinitis and asthma at 16 years. The MeDALL results have been confirmed in patient cohorts [88, 89]. It is therefore suggested that preschool children with asthma and rhinitis already sensitized may be considered as candidates for AIT.
3. *Clinical tools to evaluate AIT* There is a high degree of clinical and methodological heterogeneity among the endpoints in clinical studies on AIT, for both SCIT (sub-cutaleous AIT) and SLIT (sublingual AIT). At present, there are no commonly accepted standards for defining the optimal outcome parameters to be used for both primary and secondary endpoints [90]. A Task Force of the European Academy of Allergy and Clinical Immunology (EAACI) Immunotherapy Interest Group evaluated the currently used outcome parameters in different RCTs and provided recommendations for the optimal endpoints in future AIT trials for allergic rhinoconjunctivitis. Recommendations for nine domains of clinical outcome measures were made and recommended a homogeneous combined symptom and medication score (CSMS) was recommended as a primary outcome [90]. New ICT-based technology is likely to considerably change patient reported outcomes (PROs) for AIT during clinical trials and daily practice [6, 7, 91].
  PROs should be adapted to the age groups. Caregivers are less able than children to accurately assess response to treatment with available tools. A simple pediatric-specific tool to assess efficacy in allergic rhinitis trials in children is needed [92–95]. In older adults, very few studies have investigated PROs, but visual analogue scales appear to be of interest [92, 96, 97].
4. *Stratification of patients allowing precision medicine* Precision medicine is a medical model aiming to deliver customized healthcare—with medical decisions, practices, and/or products tailored to the individual patient. AIT has a unique immunological rationale, since the approach is tailored to the specific IgE sensitization of an individual. It modifies the natural course of the disease as it aims to have a persistent efficacy after completion of treatment. In this perspective, AIT can be considered a prototype of precision medicine [98]. Biomarkers associated with e-health, combined with a clinical decision support system (CDSS), might change the scope of AIT.
5. *Relevant immune mechanisms* Several immune changes have been associated with AIT. However, the most relevant immune mechanisms leading to clinical successful AIT have not yet been identified. This might be due to different administrations of AIT, different allergens, different products, etc. Deciphering the relevant immune mechanisms is of the upmost importance to further improve treatment protocols, finding relevant biomarkers, and to improve the composition of AIT products.
6. *Biomarkers* Biomarkers in allergic diseases and asthma are of great importance and a large body of research has been initiated [98]. The identification of biomarkers is based on systems biology approaches combining transcriptomics, proteomics, epigenetics and metabolomics in large patient cohorts. Two EU-funded projects are currently ongoing: U-BIOPRED (IMI) in severe asthma [99–101] and MeDALL (FP7) in allergy [28, 29]. These projects will help to identify biomarkers for AIT efficacy [102–104] and safety, and to better establish the personalized medicine approach [105]. The following issues should be addressed (Table 1):
7. *IgE sensitization profiles* Combinatorial analysis has shown that the IgE sensitization profiles to grass pollen in children with hay fever are very heterogeneous at the molecular level [34, 106, 107]. This high heterogeneity is important in a scenario of precision medicine. Accordingly, a classification of different categories of match and mismatch between the molecular profiles of IgE sensitization and the molecular profile of an AIT preparation has been illustrated [106]. Studies are needed to evaluate the putative impact of matched or mismatched IgE between the sensitization profile and the molecular composition of the AIT preparation used in the individual patient. Moreover, recent studies in over 1300 Italian children with seasonal allergic rhinitis have shown the importance of component-resolved diagnosis (CRD) on the prescription of AIT against pollens [108]. Molecular diagnosis with marker allergens is nowadays a promising diagnostic step in poly-sensitized individuals and it is essential to distinguish the primary sensitizers and “true” reactions from cross-reactivity caused by pan-allergens.
  AIT efficacy may be predicted by the IgE profile [102, 109, 110], but more data are needed.
8. *AIT in older adults* The role of AIT for the treatment of allergic diseases in old age should be considered. The research agenda must include not only well powered randomized controlled trials in this age group but also specific research on the immune response, time of onset of allergic disease (since childhood, adult life or late-onset in life), comorbidities, safety and cost-benefits of AIT.
9. *AIT in pregnancy* Allergic diseases such as asthma and allergic rhinitis constitute a significant burden among women of childbearing age and those who are pregnant. The continuation of AIT during pregnancy appears safe [111]. Available studies do not however show a convincing reduction in the development of atopy in offspring from the administration of AIT during pregnancy [111].
10. *AIT for skin allergy* A Cochrane review found limited evidence that AIT may be effective for people with atopic eczema [112]. The treatments used in these trials were not associated with an increased risk of local or systemic reactions. Future studies should use high quality allergen formulations with a proven track record in other allergic conditions and should include PROs as key outcome measures.
11. *SCIT versus SLIT* Direct head-to-head comparisons of subcutaneous AIT (SCIT) versus sublingual AIT (SLIT; tablets or drops) are limited [113] and have generated contradictory results [114]. Similarly, indirect comparisons are inconsistent. This is perhaps due to the lack of standardization of clinical outcomes measured, dosages, heterogeneous compositions, schedules and duration of treatment [67]. There are very few SCIT studies that meet the CONSORT criteria for publication and most are not ITT analyses [115, 116]. Moreover, efficacy and safety have been shown only for some SLIT and SCIT products. Comparisons should also be made between products [117]. For some allergen sources and age groups, effective SLIT or SCIT doses have not been established [69].
12. *AIT in polysensitized individuals* Although AIT has proven efficacy in large, robust clinical trials in primarily polysensitized patients [55, 69], more work is required to determine whether single-allergen source and multi-allergen source AIT also produce distinct immune and clinical responses in monosensitized and polysensitized patients [118]. The currently available literature suggests that when the clinically relevant allergen has been identified, AIT is effective also in polysensitized patients [118].
13. *Ideal schedule for sublingual AIT* For some allergen extract preparations, efficacy (SLIT with pollen) has been shown with a pre-coseasonal administration schedule, as opposed to other extracts for which the year-round perennial schedule is recommended. This has implications for the cost-benefit analysis as well as for adherence. Until direct comparative studies between dosing are conducted, it is speculative as to which schedule adherence might ultimately be enhanced.
14. *Duration of AIT* International harmonisation on the criteria used to determine the duration of AIT is necessary. Adherence to treatment, route of administration, cost, allergen source, patient’s comorbidities and polysensitisation are all factors that should be considered as well as the age of onset of AIT. It is usually recommended to perform AIT for a duration of 3 years, but data are insufficient.
15. *Cost*-*effectiveness* A health technology assessment study funded by the UK National Institute for Health Research Health Technology Assessment (NIHR HTA) programme concluded that a benefit from both SCIT and SLIT compared with placebo has been consistently demonstrated. However, the extent of this effectiveness in terms of clinical benefit is unclear. Both SCIT and SLIT may be cost-effective compared with AIT from around 6 years (threshold of £20,000–30,000 per QALY) [80]. In a US study based on Florida Medicaid data on allergic rhinitis patients from poverty environments, the cut-even point for SCIT was already reached at 3 months [119]. The results of this study are restricted to the US model and the population treated. Further research is urgently needed to establish the comparative effectiveness of SCIT compared with SLIT and to provide more robust cost-effectiveness estimates [71, 80]. Although it is not accepted by regulators yet, cost-effectiveness might be best shown in more real-life trial designs.
16. *Novel forms of AIT* Recombinant allergens [74, 120, 121] or the chemical and molecular modification of allergens and the use of different routes of administration of AIT should aim to facilitate its use for a wider scope of allergic diseases. However, these new options should be assessed in the context of the patient’s convenience and adherence to treatment, long-term efficacy, disease-modifying effects and cost-benefits. Biologics added to AIT may be effective for some (anti-IgE [122–124]) but not all targets (anti-IL-4 [125]). In this case, an economic evaluation is needed to stratify the patients.

## Proposal to integrate AIT in AIRWAYS ICPs

### Strategic framework

Integration and dissemination of international and national clinical practice guidelines are part of the process of ICPs. ICPs differ from practice guidelines as they are utilized by a multidisciplinary team and focus on the quality and coordination of care. Clinicians are free to exercise their own professional judgment as appropriate. An ICP is intended to act as a guide to treatment for the single patient. Any alteration to the practice identified within the ICP algorithm should be noted as a variance. Variance analysis can be used to amend the ICP itself if, for a group of patients, the practice consistently differs from the pathway.

ICPs form all or part of the clinical record, document the care given, and facilitate the evaluation of outcomes for continuous quality improvement. They should empower patients and their health and social care givers.

### Objectives

The aims of AIRWAYS ICPs are to:

- Better understand the role of AIT across the life cycle, particularly in preschool children (prevention and treatment) and in the elderly.
- Increase the awareness of the impact of AIT across the life cycle to promote AHA.
- Better stratify patients who benefit the most from AIT in all age groups.
- Launch a collaboration to develop care pathways for chronic respiratory allergic diseases integrating AIT in European countries and regions in the frame of AIRWAYS ICPs.
- Follow and implement actions and plans suggested by this integrated collaboration.
- Provide evidence for regulatory decisions.
- Propose new policies in the EU. AIRWAYS ICPs and AIT to be incorporated in Action Plan B3 of the EIP on AHA.
- Follow and implement actions and plans suggested by this integrated collaboration, which are to be endorsed by national health authorities.

### Approach

This project aims to synergize current initiatives in AIT, allergic diseases and healthy ageing, as part of the AIRWAYS ICPs programme.

*A multi*-*sectoral approach* is proposed. It will include all targeted groups such as (1) the general population (patients, parents, teachers, media, patient’s associations), (2) primary care (general practitioners and general pediatricians, pharmacists, nurses) and (3) secondary care (allergists, organ and other allergy specialists including pediatrics, ENT, internal medicine, dermatology, respiratory, clinical immunologists, and patient associations).

*Partners* will include (1) relevant academic consortia (MeDALL, GA^2^LEN, ARIA, PRACTALL), (2) relevant academic societies (both national and international), (3) manufacturers of allergy diagnostics and allergen preparations.

*A stratification of patients across the life cycle* using novel biomarkers, e-health and CDSS.

The integration of *modern e*-*health tools* should be promoted. Indeed, the use of electronic information and communications technologies in health is rising rapidly in the developing world, offering essential improvements in the management of respiratory allergies. This technology will allow better disease control and monitoring, promoting AIT compliance. These will include: (1) the Sentinel Network of GA^2^LEN and MACVIA-LR, (2) the MASK model of MACVIA-LR, including novel CDSS [7, 126] and (3) the currently available applications of certain investigators and pharmaceutical companies.

A *stepwise framework* is favoured. The AIRWAYS ICPs group will collect data, make proposals and then disseminate and evaluate actions.

- *Step 1*—*Data collection* We are aiming to create a portfolio providing all available current published data on the subject. As an example, the most recent documents provided by EAACI (AIT guidelines and consensus reports), ARIA (rhinitis and asthma guidelines), and WAO (SLIT guidelines) will be included and deposited in the EIP on AHA repository.
- *Step 2*—*Proposals and actions* We will summarize and select the best feasible proposals to integrate AIT in AIRWAYS ICP across the EU. As an example, the best windows of intervention for AIT in children and all possible interventions later in life will be scrutinized.
- *Step 3*—*Dissemination and follow*-*up* Use of the proposals will be promoted as much as possible and surveyed. As an example, a module will be created for presentations at national and EU meetings, as well as for e-learning.
- *Step 4*—*Changes in policies* Policies for senior citizens in countries should reflect the epidemiologic wave of the still unrecognized allergic diseases in the elderly. Novel approaches in health systems and reimbursement policies are needed for AIT in well-stratified patients using modern technologies. The effectiveness, cost-effectiveness and safety of such policies will need to be established.

## Abbreviations

AHA: active and healthy ageing
AIRWAYS ICPs: integrated care pathways for airway diseases
ARIA: allergic rhinitis and its impact on asthma
AIT: allergen immunotherapy
CDSS: clinical decision support system
CONSORT: consolidated standards of reporting trials
COPD: chronic obstructive pulmonary disease
CRD: chronic respiratory diseases
DG: Directorate General
EIP on AHA: European Innovation Partnership on active and healthy ageing
EU: European Union
GA^2^LEN: Global Allergy and Asthma European Network (FP6)
GARD: WHO global alliance against chronic respiratory diseases
ICP: integrated care pathway
IL: interleukin
MACVIA-LR: contre les MAladies Chroniques pour un VIeillissement Actif (fighting chronic diseases for active and healthy ageing)
MASK: MACVIA-ARIA Sentinel NetworK
MeDALL: Mechanisms of the Development of ALLergy (FP7)
MOH: Ministry of Health
NCD: non-communicable disease
PRACTALL: PRACTicing ALLergology
PRO: patient reported outcome
RCT: randomized control trial
SCIT: subcutaneous immunotherapy
SLIT: sublingual immunotherapy
Th: T helper cell
U-BIOPRED: Unbiased BIOmarkers in PREDiction of respiratory disease outcomes
WHO: World Health Organization
